# FishSegSSL: A Semi-Supervised Semantic Segmentation Framework for Fish-Eye Images

**DOI:** 10.3390/jimaging10030071

**Published:** 2024-03-15

**Authors:** Sneha Paul, Zachary Patterson, Nizar Bouguila

**Affiliations:** Concordia Institute for Information Systems Engineering (CIISE), Concordia University, Montreal, QC H3G1M8, Canada; zachary.patterson@concordia.ca (Z.P.); nizar.bouguila@concordia.ca (N.B.)

**Keywords:** semi-supervised learning, semantic segmentation, fish-eye images, autonomous driving

## Abstract

The application of large field-of-view (FoV) cameras equipped with fish-eye lenses brings notable advantages to various real-world computer vision applications, including autonomous driving. While deep learning has proven successful in conventional computer vision applications using regular perspective images, its potential in fish-eye camera contexts remains largely unexplored due to limited datasets for fully supervised learning. Semi-supervised learning comes as a potential solution to manage this challenge. In this study, we explore and benchmark two popular semi-supervised methods from the perspective image domain for fish-eye image segmentation. We further introduce FishSegSSL, a novel fish-eye image segmentation framework featuring three semi-supervised components: pseudo-label filtering, dynamic confidence thresholding, and robust strong augmentation. Evaluation on the WoodScape dataset, collected from vehicle-mounted fish-eye cameras, demonstrates that our proposed method enhances the model’s performance by up to 10.49% over fully supervised methods using the same amount of labeled data. Our method also improves the existing image segmentation methods by 2.34%. To the best of our knowledge, this is the first work on semi-supervised semantic segmentation on fish-eye images. Additionally, we conduct a comprehensive ablation study and sensitivity analysis to showcase the efficacy of each proposed method in this research.

## 1. Introduction

In the rapidly evolving landscape of computer vision applications, various real-world computer vision applications (e.g., autonomous driving) are benefiting from the use of large field-of-view (FoV) cameras [1,2]. These types of cameras use fish-eye lenses, also known as wide-angle lenses, to capture a broad view by using extensive non-linear mapping instead of regular perspective projection. However, this leads to radial distortion in the captured images, causing stretching and information loss at the edges, ultimately resulting in a blurry effect. One potential solution to the distortion issue is to use transformation techniques to convert fish-eye images into regular perspective images [3]. However, the conversion unwraps the image, resulting in the loss of a portion of the image at the edge, which ultimately compromises the rich information contained at the boundary of the images. As a result, the detection process is hindered. Another possible solution is to use the distorted fish-eye images directly in the model, as suggested by some existing research [4,5]. However, the current literature on computer vision is not well optimized for learning from fish-eye cameras, thus providing a need for further research in this area.

Semantic segmentation, an important computer vision task, focuses on classifying each pixel in an image and grouping the pixels belonging to a specific object [6]. Thus, it segments a scene into bounding areas or masks containing different objects, facilitating a proper understanding of the scene. The significance of semantic segmentation in autonomous driving lies in its ability to enable the vehicle’s sensor systems to precisely comprehend and differentiate various objects and areas in the surroundings [7]. It leads to well-informed decisions based on specific information about the road, obstacles, pedestrians, traffic signs, and other crucial elements, ultimately ensuring safer and more reliable autonomous navigation. While semantic segmentation is well explored for 2D perspective images [8], point cloud [9], depth images [10], and thermal images [11], it is relatively less explored in the context of fish-eye images [12].

There is limited research on fish-eye image segmentation that has delved into diverse methods for converting the radial distortion in fish-eye images into perspective images [3]. Some research focused on training the model on synthetic fish-eye images obtained by applying different fish-eye transformations to the perspective images [13,14]. However, these approaches do not perform well when using real-world fish-eye data. Some of the more recent works on semantic segmentation of fish-eye images focus on multi-modal, multi-task learning [1,4] to take advantage of additional information from different modalities and tasks. Nonetheless, multi-modal or multi-task data are hard to collect and annotate in large quantities to further improve the performance of the model. On the other hand, these models are computationally expensive due to the high number of learnable parameters. To this end, a single-task, uni-modal model is desirable for fish-eye image segmentation. In addition, the current literature on semantic segmentation focuses on supervised learning [3,13,15], which requires a large amount of labeled data to train deep learning models effectively. Since semantic segmentation involves classifying each pixel in an image, annotation is more costly and laborious than other computer vision tasks, such as object classification [16]. Consequently, there is a lack of publicly available semantically annotated fish-eye segmentation datasets. Semi-supervised learning (SSL) has emerged as a potential solution to this problem, allowing models to learn from a small amount of labeled data while leveraging a larger amount of unlabeled data. The basic idea of the most effective forms of semi-supervised methods involves predicting the pseudo-label for the unlabeled sample and learning from the predicted pseudo-label in an unsupervised learning setting. To the best of our knowledge, there is no existing work on SSL-based semantic segmentation for fish-eye data.

In this work, we focus on learning semantic segmentation from real-world fish-eye data in a semi-supervised setting. First, we explore and adapt various existing SSL methods from the perspective image domain, such as Mean Teacher [17] and Cross-Pseudo-Supervision (CPS) [18]. However, these methods are not originally optimized for the fish-eye data and, therefore, perform poorly. To this end, we propose a new semantic segmentation method for fish-eye images named FishSegSSL that learns effective representation from uni-modal segmentation data (single task) and improves the performance over the existing methods, including fully supervised, MeanTeacher, CPS, and CPS with CutMix. More specifically, our method incorporates into the best existing method (CPS) three key concepts that are inspired by the semi-supervised literature for image classification: pseudo-label filtering, dynamic thresholding, and robust strong augmentation. Pseudo-label filtering includes the concept of filtering low-confidence pseudo-labels to reduce the noisy signal on the unsupervised learning component. Dynamic thresholding further improves pseudo-label filtering by introducing the concept of adaptive thresholding based on the learning difficulty of different classes on the dataset. Finally, the robust strong augmentation concept introduces an additional regularizer on the easy samples so that the model does not overfit them and continues learning from all samples as the training progresses.

While there has been some research on the WoodScape datasets, existing works are either (1) fully supervised, requiring a large amount of labeled data, or (2) multi-modal or multi-task models, requiring additional data modality or annotation. To the best of our knowledge, this is the first work on fish-eye image segmentation in a semi-supervised setting. In this work, we make two main contributions in terms of novelty: (1) we present a novel (first) semi-supervised learning method specialized in fish-eye image segmentation, and (2) we propose a method that can learn from fish-eye images only, without requiring multi-modal or multi-task data. We evaluate our proposed method on the WoodScape dataset [1], which consists of real-world fish-eye images. Our extensive study shows the effectiveness of our proposed method. We also perform an in-depth ablation and sensitivity analysis on different modules of our proposed methods. Our experimental analysis shows a 10.49% improvement over fully supervised learning with the same amount of labeled data by applying the proposed method. Overall, we make the following key contributions to this work:

- We present a comprehensive study on fish-eye image segmentation by adopting conventional semi-supervised learning (SSL) methods from the perspective image domain. Our study reveals the suboptimal performance of such methods since they were not originally designed for fish-eye images.
- We propose a new SSL-based semantic segmentation method that incorporates three key concepts: pseudo-label filtering to reduce noisy pseudo-labels, dynamic thresholding for adaptive filtering based on class difficulty, and robust strong augmentation as a regularizer on easy samples.
- This is the first work on semi-supervised fish-eye image segmentation that is uni-modal and single-task.
- We perform an extensive experimental analysis with a detailed ablation and sensitivity study on the WoodScape dataset, revealing a 10.49% improvement over fully supervised learning with the same amount of labeled data and showcasing the efficacy of the introduced method.
- We make the code available at: https://github.com/snehaputul/FishSegSSL (accessed on 15 January 2024).

The subsequent sections of the paper are structured as follows: Section 2 reviews prior research pertinent to our proposed model. Section 3 provides an in-depth presentation of the proposed method. Section 4 and Section 5 delve into the discussion of the experimental results. Finally, Section 6 concludes with closing remarks and considerations for the future direction of this research.

## 2. Related Works

In this section, we will discuss the available work in semantic segmentation on regular perspective and fish-eye images in other relevant domains. Then, we will elaborate on the existing literature on semantic segmentation models for autonomous driving.

### 2.1. Perspective Image Segmentation

Semantic segmentation is a popular dense prediction task in computer vision that has seen significant developments in the past few years, including CNN- and vision transformer (ViT)-based models. Vision transformers are gaining popularity in semantic segmentation for the advantage gained from their special network architecture. These models learn pixel-level feature representation using a transformer-based encoder, an attention mechanism, and a bottle-neck layer [19,20]. SegViT [21] stands apart from conventional ViTs because it uses an Attention-to-Mask (ATM) module. This module effectively transfers learned similarity maps, derived from trainable parameters and spatial feature maps, into segmentation masks. They also propose a shrunk structure of the SegViT model based on a query-based up-sampling (QU) and query-based down-sampling (QD) module that reduces a model’s computational cost by 40%. SegFormer [22] is another ViT-based semantic segmentation model that incorporates a transformer-based encoder with a lightweight full MLP-based decoder. SegFormer does not require positional encoding that needs to be interpolated, resulting in reduced performance of the model when the resolution of training and testing data is different. Thus, it introduces Mix-FFN, which is a combination of convolution blocks with MLP that can still capture spatial information without positional encoding. However, none of these SOTA models have been explored in the concept of fish-eye image segmentation.

### 2.2. Fish-Eye Image Segmentaiton

In recent years, there has been a bit of research on fish-eye image segmentation that addresses distortion at the edge of the image. One such approach, proposed in [16], uses an Efficient Residual Factorized Network (ERFNet)-based architecture to handle distortion in synthetic fish-eye data. Another approach, proposed in [15], uses an adaptive deformable CNN-based architecture that can adapt to fish-eye images while being trained on pinhole camera images, thus avoiding the laborious task of data transformation. However, due to the lack of large public fish-eye datasets, most of the existing literature converts regular perspective images into fish-eye images. In [23], the method focused on the development of a rotation-invariant multinet for fish-eye images in the context of autonomous driving applications. In [24], the method focused on the augmentation of viewpoint for image semantic segmentation using surround-view fish-eye cameras. Additionally, Ref. [25] presented a self-supervised scale-aware distance estimation method utilizing monocular fish-eye cameras for autonomous driving. Ref. [26] reports the result of various supervised methods for fish-eye image segmentation by leveraging large labeled data arranged through a competition. In [14], a method is proposed to reuse rectilinear images as fish-eye images to address this issue. In [13], a seven-degree-of-freedom (DoF) augmentation method is proposed, which uses rectilinear images during training to transform them into fish-eye images. This method simulates fish-eye images from rectilinear images captured from different cameras with varying focal lengths, positions, and orientations. Although the seven DoF augmentation method is effective in increasing the model’s mean intersection over union (mIoU) compared to highly distorted real-world fish-eye data, it falls short in situations where the distortion is not similar among images, which is a common criterion in real-world scenarios. As a result, existing models trained on transformed or synthetic fish-eye data do not achieve superior performance when using real-world fish-eye data. Another key observation we find is that the very recent literature is based on multimodal or multitask models where the signals from different tasks or domains help the model to learn representation from fish-eye images [4,27]. However, such a dataset is costly to collect and computationally demanding to train. Therefore, our research focuses on learning semantic segmentation from real-world fish-eye data using the image alone.

### 2.3. Segmentation for Autonomous Driving

Various existing works deal with semantic segmentation for autonomous driving scenarios from supervised settings. For example, [28,29] worked on multi-class semantic segmentation, whereas [30] focused on road segmentation. Ref. [31] proposed a fully convolutional network (FCN)-based architecture, a one-stage pipeline using fully connected layers to replace the convolutional layers for predicting coarse output maps. These coarse maps are up-sampled by deconvolution to produce dense pixel-level labels. Ref. [29] extended this idea by using an encoder–decoder-based CNN network. However, these networks work in supervised settings, where labeled data is necessary, making semantic segmentation a laborious and costly task.

To this end, a couple of studies on unsupervised settings have been proposed, requiring no [32] or little [33] labeled data. The Asynchronous Teacher-Student Optimization (ATSO) algorithm [34] used continual learning by partitioning unlabeled training data into two subsets. One subset of the training data was used for fine-tuning the model that updated the labels of the other subset. Ref. [35] proposed a semi-supervised perspective image segmentation network using adversarial learning. Ref. [36] proposed a multi-task, self-supervised segmentation network that is efficient for classification, segmentation, and detection tasks when labeled data are unavailable. Though these models perform satisfactorily on perspective images, their performance deteriorates for fish-eye images due to high radial distortion. Thus, an in-depth study of the unsupervised segmentation domain for fish-eye autonomous driving images is necessary.

In summary, the current research on semantic segmentation has primarily focused on different modalities, excluding fish-eye images, particularly in situations where labeled data are scarce and annotation is expensive and time-consuming. Moreover, a noticeable gap exists in studies conducted on real-world fish-eye images captured from autonomous vehicles (AVs). To this end, our work introduces a unimodal SSL-based semantic segmentation framework specifically designed for real-world fish-eye images.

## 3. Method

In this section, we present details of our methodology. First, we discuss the preliminaries of semi-supervised learning and the existing methods that we adopted in our study. Then, we discuss our proposed SSL components to address the existing research gap in the semi-supervised fish-eye image segmentation literature.

### 3.1. Priliminaries

In this work, we explore two well-known semi-supervised segmentation methods and one of their variants that was originally developed for the perspective image domain: MeanTeacher [17], CPS [18], and CPS with CutMix augmentation. The following is a short description of all methods in our study.

MeanTeacher [17] is a semi-supervised learning method that involves two encoders: an online encoder and a teacher encoder. Inspired by the semi-supervised literature of object recognition [37], it updates the teacher encoder as an exponential moving average (EMA) of the online encoder (student encoder), denoted as $\bar{\theta }$. The model is trained using a consistency regularization task. The consistency regularization works in a way to align the predicted pseudo-label $Z_{1}$ of the student network to the feature map $Z_{2}$ of the teacher network. The loss function of MeanTeacher can be represented as:

(1) $$\begin{array}{cc} X & \to X_{1}\to f\left(\theta \right)\to Z_{1}\\ \; & ↘X_{2}\to f\left(\bar{\theta }\right)↛Z_{2} \end{array}$$

where $X_{1}$ and $X_{2}$ are two different augmentations of images of an input image *X*, and θ is the parameter of the model. At training time, $Z_{1}$ is supervised by $Z_{2}$. However, the teacher network does not back-propagate the loss but is updated as the student network’s exponential moving average (EMA). The EMA helps to stabilize the student model’s updates, smoothing out fluctuations and facilitating knowledge transfer to the student network. This technique enhances the model’s capacity to handle unlabeled data and improves overall performance on both labeled and unlabeled datasets. It reduces the redundant calculation and training cost while keeping the teacher network weights similar to the student network.

Cross-Pseudo-Supervision (CPS), introduced in [18], is a novel SSL method that concurrently leverages information from both labeled and unlabeled perspective images by deploying consistency regularization. CPS aims to increase the consistency between two different segmentation networks for similar input images. Both labeled and unlabeled data are fed into the two different networks, and for each unlabeled image, the model generates a one-hot pseudo-prediction map. This pseudo-prediction map is then used to supervise the other segmentation network and vice versa. This way, the pseudo-segmentation map is used as an additional signal to provide supervision for the unlabeled data. Formally, CPS generates the prediction as follows:

(2) $$\begin{array}{cc} Z_{1} & =f(X;\theta_{1}), \end{array}$$

(3) $$\begin{array}{cc} Z_{2} & =f(X;\theta_{2}). \end{array}$$

Here, $Z_{1}$ and $Z_{2}$ are the segmentation predictions obtained from the two segmentation networks after applying the softmax function. Note that the two segmentation networks are similar in structure but initialized and updated differently. The predictions are then used to generate the pseudo-labels. The whole process can be described as follows:

(4) $$\begin{array}{cc} \; & ↗f\left(\theta_{1}\right)\to Z_{1}↛{\hat{Y}}_{1}\\ X & \;\;\;\;\;\;\;\;\;\;\;\;\;\;\;\;\;\;\;\;↖\;↙\\ \; & ↘f\left(\theta_{2}\right)\to Z_{2}↛{\hat{Y}}_{2}, \end{array}$$

where ${\hat{Y}}_{1}$ and ${\hat{Y}}_{2}$ are one-hot pseudo-labels.

The CPS method imposes two loss functions: a supervised loss and a pseudo-supervised loss (unlabeled loss). The supervised loss is a standard cross-entropy loss applied at the pixel level. The pseudo-supervised loss (unsupervised loss) is also a standard cross-entropy loss applied bidirectionally on the unlabeled data and predicts pseudo-labels. The overall cross-pseudo-supervised loss is the sum of the supervised loss on the labeled data and the pseudo-supervised loss on the unlabeled data.

**Figure 1:**
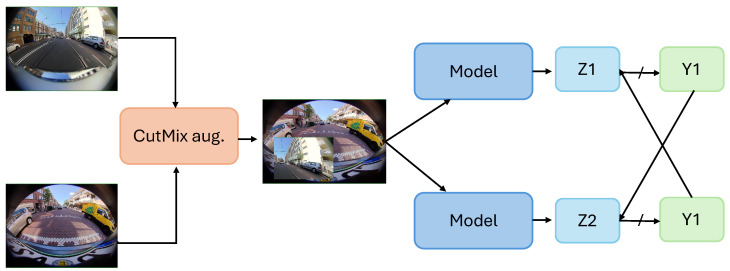
An overview of the Cross-Pseudo-Supervision (CPS) method. Here, $Z_{1}$ and $Z_{2}$ are the softmax incorporated segmentation predictions, and $Y_{1}$ and $Y_{2}$ are one-hot pseudo-labels. ↛ shows no flow of gradient.

CPS also introduces a variant where it utilizes CutMix augmentation between two images before feeding it to the model. Figure 1 represents an overview of the CPS method with CutMix augmentation. In the CutMix augmentation, a portion of the image is cropped and cut, and then the cropped portion of the image is mixed with another image, creating a diversified sample set. This augmented image is fed as input in both of the networks $f(\theta_{1})$ and $f(\theta_{2})$. The augmentation provides a regularization effect, making learning from data challenging, thus reducing overfitting.

### 3.2. Proposed Method

**Figure 2:**
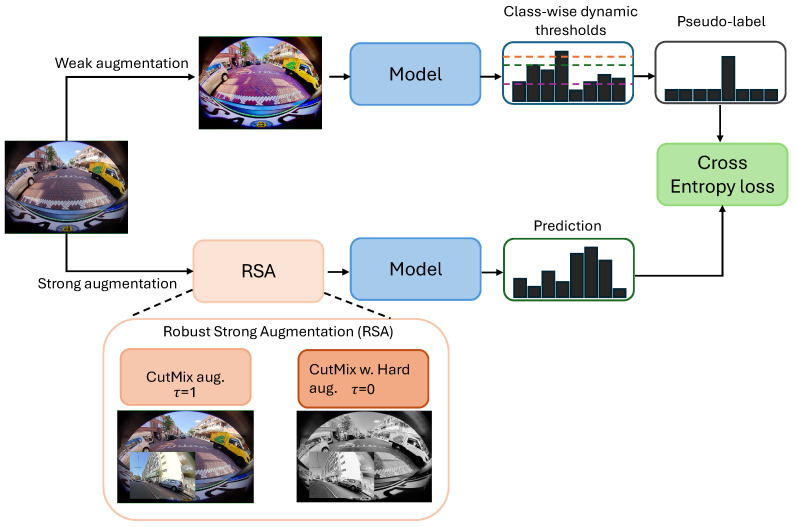
An overview of our proposed semi-supervised fish-eye image segmentation (FishSegSSL) framework. Here, RSA represents the robust strong augmentation module. If the threshold τ=1, only the CutMix augmentation is applied to the input image. For τ=0, the RSA module is activated by combining CutMix augmentation with hard augmentation. The three colored lines are examples of class-wise dynamic confidence thresholds for three different classes. The pseudo-label filtering mechanism utilizes dynamic confidence thresholding; finally, a cross-entropy loss is applied to the prediction and the pseudo-label.

In this section, we discuss our proposed semi-supervised framework for fish-eye image segmentation by introducing three components to the existing best-performing method (illustrated in Figure 2). The three SSL components, which aim to improve semi-supervised segmentation on fish-eye images, are described in the following subsections.

#### 3.2.1. Pseudo-Label Filtering

While the CPS method utilizes all predicted pseudo-labels for learning, it also incorporates predictions that are not correct. To deal with this issue, we incorporate the concept of confidence thresholding that reduces low-confidence prediction, effectively removing most of the wrong predictions. It is shown in the context of the image classification literature on SSL [38] that such filtering can reduce the noise in the learning signal from unlabeled data and improve performance. In the pseudo-label filtering, a weakly augmented image is first fed as input to the model f(θ), which predicts the pseudo-labels $Z_{w}$ that are transformed into one-hot pseudo-labels and ${\hat{Y}}_{w}$ if the confidence of the prediction is greater than a threshold (τ). The same image is then subjected to strong augmentation and fed into the same model, f(θ). The one-hot pseudo-label from the weakly augmented image, f(θ), is used to supervise the predictions of the strongly augmented image, $Z_{s}$. This process works as a consistency regularizer by ignoring low-confidence pseudo-labels for supervision. Incorporating the pseudo-label filtering, our learning task can be represented as follows:

(5) $$\begin{array}{cc} \; & X\to X_{s}\to f\left(\theta \right)\to Z_{s}\\ \; & ↓\;\;\;\;\;\;\;\;\;\;\;\;\;\;\;\;\;\;\;\;\;\;\;\;\;\;\;\;↖\\ \; & X_{w}\to f\left(\theta \right)\to Z_{w}\left(>\tau \right)↛{\hat{Y}}_{w}, \end{array}$$

where $X_{w}$ and $X_{s}$ are the weak and strong augmentations of image *X*. The pseudo-label filtering component is combined with the existing best-performing method, i.e., CPS with CutMix augmentation.

#### 3.2.2. Dynamic Confidence Thresholding

While pseudo-label filtering uses a fixed threshold value to filter out high-confidence pseudo-labels, this results in a class-wise inconsistent learning status. Since different classes have different levels of difficulty, the thresholding mechanism should be different according to the difficulty of the classes. Inspired by [39], we incorporate a dynamic thresholding strategy with pseudo-label filtering based on the class-wise learning status. We define the learning status as the percentage of samples being correctly predicted with high confidence by the model. Depending on this learning status for each class, the threshold value is adjusted and calculated in each epoch. More specifically, we calculate the class-wise learning status and the confidence threshold as follows:

(6) $$\gamma_{t}\left(n\right)=\frac{\alpha_{t}\left(n\right)}{\max_{n}\alpha_{t}},$$

(7) $$Y_{t}\left(n\right)=\gamma_{t}\left(n\right)\cdot \tau$$

Here, $\alpha_{t}\left(n\right)$ represents class-wise learning status for *n* class at *t* time. $Y_{t}\left(n\right)$ is the normalization of $\alpha_{t}\left(n\right)$ obtained by dividing by the maximum learning status of a class. The normalization is performed to scale this value between 0 and 1. $\alpha_{t}\left(n\right)$ is then used to adjust the threshold $Y_{t}\left(n\right)$. Thus, the class with maximum learning status, i.e., the easy-to-learn class, obtains $Y_{t}\left(n\right)$ the same as τ, while the lower learning status classes, i.e., the hard-to-learn classes, achieve a $Y_{t}\left(n\right)$ that is lower than τ. Finally, the pseudo-label filtering module utilizes the dynamic threshold values to filter high-confidence pseudo-labels.

#### 3.2.3. Robust Strong Augmentation

Although the previous literature has explored the combination of CPS and CutMix augmentation, the effectiveness of augmentation in the context of SSL still remains unexplored. Augmentation serves a regularization purpose, creating modified versions of existing data to expand the training set, thereby improving the model’s performance and mitigating the risk of overfitting. However, confidently classified augmented samples indicate that the model has already learned effectively from them, thus resulting in loss values that are close to zero, effectively providing no further learning signal. These samples offer no benefits to the model’s learning process. Inspired by [40], we introduced a robust strong augmentation module to further utilize these samples. This module selects high-confidence samples by computing a historical loss in each *t*-th epoch, shown in Equation (8). This historical loss, $L_{historic}$, essentially represents a consistency loss, continually updated through the exponential moving average (EMA), along with its parameters. The equation for historical loss is given below:

(8) $$L_{historic}^{t}=\left(1-\beta \right)L^{t-1}+\beta l^{t}$$

The historical loss, $L_{historic}$, identifies high-confidence samples where there are always samples correctly classified by the model, resulting in low consistency loss over time. Depending on this historical loss, a threshold segmentation function, OTSU [41], is used to calculate the historical loss threshold, γ, for each epoch *t*. γ is used to identify low- and high-confidence samples, and a hard augmentation, which is a combination of different augmentations, e.g., brightness and color adjustment and color jitter, is used over the CutMix augmentation to make further varied samples. This is represented by the following equations:

(9) $$\mathrm{Augmented sample}\left(x_{i}\right)=\left\{\begin{array}{c} R\left(x_{i}\right),\gamma_{i}=1\\ R^{\prime }\left(x_{i}\right),\gamma_{i}=0 \end{array}\right.$$

(10) $$R^{\prime }\left(x_{i}\right)=\mathrm{Concat}\left(R\left(x_{i}\right),\mathrm{hard augmentation}\right)$$

Here, $R^{\prime }\left(x_{i}\right)$ represents the robust strong augmentation module, which is the contamination of CutMix augmented samples with hard augmented samples. When the historical loss threshold, γ, is 1, only CutMix augmentation, $R(x_{i})$, is applied on the unlabeled samples $x_{i}$ and vice versa. It is worth noting that it is critical for the interchangeability of the hard augmentation and CutMix augmentation to occur in a timely manner, as excessive augmentation can potentially harm the model by introducing excessive perturbations to the samples.

The adaptive augmentation module can be used in a plug-and-play manner with any method. Thus, we incorporate this module with our proposed pseudo-label filtering and dynamic thresholding strategy with CPS and CutMix augmentation. The ablation studies are discussed in Section 4.4.

## 4. Experiments

This section presents the results of this study. First, we will discuss the dataset and implementation details. Then, we will discuss the performance of existing methods in fish-eye image segmentation, which we consider the baseline of this study. Finally, we will present a discussion of the results of our proposed methods.

### 4.1. Dataset and Implementation Details

**Dataset Description.** WoodScape [1] is a dataset specifically designed for autonomous driving tasks captured using multi-camera fish-eye lenses. It comes with extensive ground truth annotations for nine essential tasks, including semantic segmentation, which covers classes like roads, pedestrians, and traffic signs. The main purpose behind WoodScape is to offer a comprehensive platform for evaluating computer vision algorithms utilized on fish-eye images. This dataset stands out as the first labeled dataset specifically designed for semantic segmentation. The data were collected from three distinct geographical regions: the US, Europe, and China. The collection of data consists of four surround-view cameras and involves nine distinct tasks, including depth estimation, 3D bounding-box detection, and soiling detection. Overall, WoodScape promises high-quality data and opens avenues for developing unified multi-task and multi-camera models for advancing autonomous driving technology.

**Implementation Details.** We follow OmniDet [27] and SemiSeg [18] for the training setup and default hyper-parameters. The visual encoder backbone of OmniDet is a ResNet50 network. We first divide the total training samples from WoodScape [1] into supervised and unsupervised splits for semi-supervised training. More specifically, we used 20% of the 8029 samples as our labeled set and the rest of the samples as an unlabeled set. We trained the model for 125 epochs with an Adam optimizer, a learning rate of 0.0001, and a batch size of 16. A multi-step scheduler with a decay factor of 0.1 and a step size of [100, 110] was used for learning rate adjustment. The model was trained on an NVIDIA RTX A6000 GPU.

### 4.2. Baseline

For this and all subsequent experiments, we consider supervised learning with an equal amount of labeled data (20% to total data) used in the semi-supervised methods as the baseline. So, the improvement with the semi-supervised method is purely from using unlabeled data (as the number of labeled samples is the same in both settings). The first row of Table 1 shows the baseline mIoU for our work, which is 54.32. For the rest of the paper, we will discuss the improvement in different semi-supervised methods over this baseline.

### 4.3. Main Results

Table 1 summarizes the results for all the existing methods as well as our proposed semi-supervised training framework. We followed the protocol described in [18] for evaluation. More precisely, the evaluation process is unimodal: single-scale testing. The results are presented utilizing the mean intersection over union (mIoU) metric, which evaluates the model’s accuracy by computing the intersection-to-union ratio between predicted and ground truth masks for each class. The mIoU offers an average assessment of segmentation performance across all classes, with higher values denoting superior performance. As mentioned in the Section 3, we re-implemented three popular semi-supervised segmentation methods from the regular image literature, namely, MeanTeacher [17], CPS [18], and CPS with CutMix augmentation [18]. We found the best result for CPS with CutMix augmentation from existing methods. It obtained an mIoU of 62.47, which is an 8.15% improvement of the supervised learning with the same number of labeled samples. The next best performance was shown by the original CPS with an mIoU of 60.31. MeanTeacher did not perform well, and the result was worse than learning from supervised loss only. However, our proposed method, FishSegSSL, outperformed all previously explored methods by gaining an mIoU of 64.81. This provides an **2.34%** improvement over the existing best practice and a **10.49%** improvement over the fully supervised baseline.

### 4.4. Ablation Study

In this section, we conduct a detailed ablation study to assess the impact of our proposed components on the model’s performance. Each component is individually removed, and the mean intersection over union (mIoU) for each setting is reported.

In Table 2, in the first configuration, we incorporate all three components—pseudo-label filtering strategy, dynamic confidence thresholding, and robust strong augmentation—with the existing best-performing method, CPS with CutMix augmentation, which yields an mIoU of 64.81. Note that it is also the highest performance reported in this study. Subsequently, we excluded the dynamic confidence thresholding component, replacing it with a fixed threshold value (0.90) for pseudo-label filtering, resulting in a 1.92% drop in model performance. This underscores the superiority of class-wise confidence-based dynamic thresholds for filtering pseudo-labels over fixed threshold values.

We further removed the robust strong augmentation module while retaining the pseudo-label filtering and dynamic confidence thresholding. This resulted in lower model performance than the complete three-component setup, i.e., the first configuration of the ablation study. Nevertheless, when incorporating pseudo-label filtering alongside dynamic confidence thresholding, the model performs better than when pseudo-label filtering and robust strong augmentation are considered in isolation. This provides the interesting insight that dynamic confidence thresholding has a more substantial impact on leveraging the model than robust strong augmentation. However, it is not viable to explore a scenario where we remove pseudo-label filtering and solely consider dynamic confidence thresholding. This limitation arises because dynamic confidence thresholding adjusts the threshold value based on class-wise confidence rather than relying on a fixed threshold value. The pseudo-label filtering component utilizes this dynamic threshold to selectively filter out high-quality pseudo-labels. Isolating only the robust strong augmentation component leads to a further decline in model performance, emphasizing the efficacy of considering high-quality pseudo-labels over employing hard augmentation for easy samples. Omitting all three proposed components reverts to the best-performing existing method, CPS with CutMix augmentation, with a 2.34% decrement from our proposed FishSegSSL, demonstrating the effectiveness of our framework.

### 4.5. Sensitivity Study

In this section, we discuss the results of the sensitivity study on the existing methods that we find important for fish-eye segmentation performance.

#### 4.5.1. Hyperparameter Sensitivity

Table 3 shows different combinations of CPS weight and learning rate values for the CPS method. The table includes the performance of the CPS method for different combinations of CPS weights of 1.5, 1, and 0.5. We can see that with the change in the CPS weights with a fixed learning rate, the model’s performance does not change significantly. However, by decreasing the learning rates from 0.01 to 0.0001 with a constant CPS weight, the model’s performance increases significantly. The highest mIoU, 60.40%, is achieved with a 0.0001 learning rate with 0.5 CPS weight. So, it can be concluded that the model’s performance is more sensitive to the learning rate compared to the CPS weight.

#### 4.5.2. Confidence Thresholding

Table 4 shows the performance of CPS with confidence thresholding for different threshold values. The results indicate that the highest mIoU value of 60.66 is obtained with a threshold of 0.75, whereas the lowest value of 59.96 is obtained with a threshold of 0.9. Since the threshold value works as a consistency regularizer by ignoring low-confidence pseudo-labels, the higher threshold value induces a higher regularization effect on the model and thus affects the model’s performance by ignoring a larger amount of predicted pseudo-labels for supervision.

Table 5 demonstrates the performance of CPS with CutMix and confidence thresholding at various threshold values. We consider the threshold values of 0.5, 0.75, 0.9, and 0.95 in this study. The results indicate that the highest mIoU is achieved with a threshold of 0.5, while the lowest is observed with a threshold of 0.95. We believe the reason behind the following performance of the model is similar to that explained in the previous subsection.

To understand the performance of CPS with CutMix augmentation, shown in Table 6, we conduct experiments using different combinations of the number of CutMixed boxes and the range of these boxes. The table demonstrates the method setting and the corresponding mIoU value. This experiment aims to determine the optimal combination of box number and range that would yield the highest mIoU value. The default setting for the experiment had a box number of 3 and a range of 0.25–0.5, with an mIoU value of 62.47. The highest performance, 62.71%, is achieved by four CutMix boxes with a range of 0.4 to 0.75. Also, note that with the increment of the CutMix range, the model’s performance is enhanced as the overlapping area of the two images increases. Thus, the model obtains a wider view of the images to capture the underlying information.

## 5. Discussion

In this study, we present an adaption of traditional semi-supervised learning methods to real-world fish-eye data, exposing their suboptimal performance and lack of optimization for fish-eye characteristics. To deal with this challenge, we propose a novel SSL-based semantic segmentation method featuring pseudo-label filtering, dynamic thresholding, and robust strong augmentation. At first, we establish a supervised learning baseline with 20% labeled data, yielding an mIoU of 54.32, and subsequently compare different semi-supervised methods, identifying CPS with CutMix augmentation as the top performer with an 8.15% improvement over supervised learning.

We further propose a novel semantic segmentation framework with three SSL components inspired by the existing SSL literature in the perspective image domain. The components are pseudo-label filtering, dynamic thresholding, and robust strong augmentation modules. We then combined the proposed components with CPS and CutMix augmentation, the existing best-performing setting. The significance of the proposed pseudo-label filtering is that it ensures superior supervision by filtering high-quality pseudo-labels by leveraging a fixed threshold value or dynamic confidence thresholding. The robust strong augmentation module imposes a hard augmentation on the easy samples, creating a varied sample set and ensuring proper utilization of the unlabeled set. From experimental analysis, we can see that the dynamic threshold strategy outperforms all the existing methods by achieving a notable 2.34% improvement over existing methods and a substantial 10.49% improvement over the fully supervised baseline. We also performed detailed ablation studies to underscore the significance of the proposed components. The ablation study proves that the pseudo-label filtering strategy with dynamic confidence thresholding and robust strong augmentation outperforms alternatives. Further investigations on sensitivity analysis reveal the model’s sensitivity for both model-specific hyperparameters and threshold values. Overall, the findings showcase the effectiveness of our proposed confidence threshold and robust strong augmentation module with CPS and CutMix augmentation in significantly enhancing semi-supervised segmentation models for fish-eye images.

## 6. Conclusions

Though fish-eye images are beneficial in various real-world scenarios for capturing large fields of view (FOVs), they are not well explored due to the lack of large publicly annotated datasets. In this work, we explored semi-supervised learning as a solution to the data scarcity problem for fish-eye image segmentation, which has not been studied in the context of fish-eye images. This paper explored two existing semi-supervised methods developed on perspective images and benchmarked their performances. We then proposed a unimodal semi-supervised semantic segmentation method for fish-eye images that introduces three semi-supervised learning components: pseudo-label filtering, dynamic confidence thresholding, and robust, strong augmentation. An analysis of the proposed framework shows that semi-supervised methods can improve up to 10.49% over fully supervised methods for the same amount of labeled data. However, one of the limitations of our work is that we have focused on a subset of the available literature concerning SSL-based semantic segmentation methods designed for perspective images. As a result, we recognize the potential for enhancing the performance (mIoU) in semantic segmentation tasks for fish-eye images by delving into other SSL methods already established in the field. We hope that this work will encourage the evolution of further specialized techniques for learning semantic segmentation and other computer vision tasks related to fish-eye images, especially when labeled data are scarce.

## Figures and Tables

**Table 1:** Comparison between fully supervised learning and semi-supervised learning with unconstrained unlabeled data. The bolds indicate the superior performance of our methods.

Method	mIoU
Fully supervised (baseline)	54.32
MeanTeacher [17]	54.20
CPS [18]	60.31
CPS with CutMix [18]	62.47
**FishSegSSL (ours)**	**64.81**

**Table 2:** Ablation study of proposed SSL components, representing the effect of each proposed component on model performance. Here, PLF, RSA, and DCT represent pseudo-label filtering, robust strong augmentation, and dynamic confidence thresholding, respectively. The bold indicates the superior performance of our methods.

PLF	RSA	DCT	mIoU
✓	✓	✓	**64.81**
✓	✓		62.89
✓		✓	63.94
	✓		62.49
			62.47

**Table 3:** Comparison among various combinations of CPS weight and learning rate values for our proposed CPS with flexible thresholding with CutMix and robust strong augmentation module. The bold indicates the superior performance of our methods.

	CPS Weight		
**LR**	1.5	1	0.5
0.01	47.36	46.46	46.67
0.001	57.28	57.19	56.10
0.0001	60.31	59.86	60.40

**Table 4:** Performance of CPS with fixed confidence thresholding for different threshold values.

Threshold Value	mIoU
0.5	60.56
0.75	60.66
0.9	59.96

**Table 5:** Performance of CPS with CutMix and fixed confidence thresholding for different threshold values.

Threshold Value	mIoU
0.5	62.38
0.75	61.98
0.9	62.12
0.95	55.60

**Table 6:** Performance of CPS with CutMix augmentation with different combinations of the number of CutMixed boxes and the range of boxes of CutMix augmentation.

Method Setting	mIoU
Default (box number = 3; range = 0.25–0.5)	62.47
nb. of. box = 2 and range = 0.1 to 0.25	62.13
nb. of. box = 2 and range = 0.25 to 0.5	61.60
nb. of. box = 2 and range = 0.4 to 0.75	61.29
nb. of. box = 3 and range = 0.1 to 0.25	61.02
nb. of. box = 3 and range = 0.4 to 0.75	61.59
nb. of. box = 4 and range = 0.1 to 0.25	61.00
nb. of. box = 4 and range = 0.25 to 0.5	60.91
nb. of. box = 4 and range = 0.4 to 0.75	62.71

## Data Availability

The WoodScape dataset is publicly available at https://woodscape.valeo.com/woodscape/download (accessed on 15 January 2024).
